# Predictive machine learning models for anticipating loss to follow-up in tuberculosis patients throughout anti-TB treatment journey

**DOI:** 10.1038/s41598-024-74942-z

**Published:** 2024-10-21

**Authors:** Jingfang Chen, Youli Jiang, Zhihuan Li, Mingshu Zhang, Linlin Liu, Ao Li, Hongzhou Lu

**Affiliations:** 1 The Third People’s Hospital of Shenzhen, Shenzhen, 518112 China; 2 National Clinical Research Center for Infectious Diseases, Shenzhen, 518112 China; 3 Second Hospital Affiliated With Southern University of Science and Technology, Shenzhen, 518112 China; 4 Faculty of Medicine, Macau University of Science and Technology, Macau, 999078 China; 5 Nursing Department, The People’s Hospital of Longhua, Shenzhen, 518109 China; 6 Hengyang Medical School, School of Nursing, University of South China, Hengyang, 421001 China; 7 Clinical Nursing Teaching and Research Section, The Second Xiangya Hospital of Central South University, Changsha, China

**Keywords:** Machine learning, Tuberculosis, Loss to follow-up, Anti-TB treatment, Predictive models, Artificial intelligence, Health care, Risk factors, Mathematics and computing

## Abstract

Loss to follow-up (LTFU) in tuberculosis (TB) management increases morbidity and mortality, challenging effective control strategies. This study aims to develop and evaluate machine learning models to predict loss to follow-up in TB patients, improving treatment adherence and outcomes. Retrospective data encompassing tuberculosis patients who underwent treatment or registration at the National Center for Clinical Medical Research on Infectious Diseases from January 2017 to December 2021 were compiled. Employing machine learning techniques, namely SVM, RF, XGBoost, and logistic regression, the study aimed to prognosticate LTFU. A comprehensive cohort of 24,265 tuberculosis patients underwent scrutiny, revealing a LTFU prevalence of 12.51% (n = 3036). Education level, history of hospitalization, alcohol consumption, outpatient admission, and prior tuberculosis history emerged as precursors for pre-treatment LTFU. Employment status, outpatient admission, presence of chronic hepatitis/cirrhosis, drug adverse reactions, alternative contact availability, and health insurance coverage exerted substantial influence on treatment-phase LTFU. XGBoost consistently surpassed alternative models, boasting superior discriminative ability with an average AUC of 0.921 for pre-treatment LTFU and 0.825 for in-treatment LTFU. Our study demonstrates that the XGBoost model provides superior predictive performance in identifying LTFU risk among tuberculosis patients. The identification of key risk factors highlights the importance of targeted interventions, which could lead to significant improvements in treatment adherence and patient outcomes.

## Introduction

Tuberculosis remains a pressing global health challenge, demanding sustained follow-up and treatment adherence for successful outcomes^1^. Robust treatment adherence is crucial for the effectiveness of TB treatment^1^. Loss to follow-up (LTFU) is a significant barrier to effective TB management, leading to treatment failure, drug resistance, and increased mortality, which negatively impacts families and society^2,3^.

LTFU is a global issue, with the World Health Organization reporting an average global rate of 6%, and some countries showing rates from 4 to 38% before treatment^2,4^. While studies have reported treatment attrition rates in France (15%)^5^, India (19%)^6^, and Japan (7.8%)^7^, comprehensive data from China remain limited. Notably, diverse factors contribute to the LTFU, encompassing cultural nuances, social contexts, and geographic disparities. Moreover, the triggers for visit loss at distinct stages can exhibit considerable variability^8^.

Machine learning has become increasingly important in detecting, diagnosing, and predicting TB outcomes. Techniques like Support Vector Machines (SVM), Deep Neural Networks, and Random Forests have demonstrated utility in aiding physicians to identify tuberculosis-related lesions in medical images, thereby enhancing diagnostic precision^9^. Convolutional neural network (CNN) models have successfully analyzed chest X-ray images to detect tuberculosis lesions with greater accuracy compared to conventional physician diagnosis^10^. Neural network models have integrated patients’ clinical attributes and biomarkers to prognosticate the likelihood of drug resistance in TB patients^11^. XGBoost is particularly powerful for handling complex data and enhancing predictive accuracy, especially with large, high-dimensional datasets^12^. Logistic regression, known for its simplicity and interpretability, is often used as a benchmark to compare more complex models like XGBoost, which has shown superior predictive power in our study.

This study uses machine learning to develop predictive models for LTFU in TB patients before and during treatment. Leveraging patient data from the National Center for Clinical Research in Infectious Diseases and follow-up data from the CDC, our objective is to craft robust and efficient models that empower healthcare practitioners to make informed decisions and mitigate the challenges posed by LTFU.

## Method

### Study design and participants

This study retrospectively collected data on tuberculosis patients treated or registered at the National Center for Clinical Medical Research on Infectious Diseases between January 1, 2017, and December 31, 2021, to create the training and validation datasets for the algorithm models. Follow-up data from the National Center for Disease Control (CDC) were used to verify and develop algorithm models, including SVM, Random Forest (RF), Extreme Gradient Boosting (XGBoost), and logistic regression, to predict LTFU before and during treatment. This retrospective study used anonymized data, ensuring privacy. Ethical approval and a waiver for obtaining informed consent from the study participants were granted by the Ethics Review Committee of the Third People’s Hospital of Shenzhen (Approval Number: [2022-027]). As the study did not involve direct patient participation, informed consent was not required. All research methods adhered to relevant ethical guidelines and legal regulations.

### Definition of LTFU

LTFU, as defined by the WHO tuberculosis reporting framework^13^, includes two groups: (1) patients who did not start treatment after diagnosis; and (2) patients who started treatment but had interruptions of two or more consecutive months. Employing this definition, patients who hadno more than one treatment record were labeled as LTFU before the treatment. Meanwhile, individuals exhibiting intervals surpassing two months between consecutive medical visits subsequent to treatment initiation were categorized as LTFU during the treatment trajectory.

### Include and exclude

The following two groups were included in this study: (1) Bacteriologically confirmed TB cases: patients with positive results confirmed by smear microscopy, culture, or WHO-approved rapid diagnostic methods (e.g. GeneXpert MTB/RIF). (2) Clinically diagnosed TB cases: patients with TB who show negative results on bacteriologic tests but are diagnosed with active TB by a clinician and start a full course of treatment regimen. The exclusion criteria were (1) Cases that were regularly seen as of December 30, 2021 and have not yet finished treatment; (2) Cases registered as dead and treatment failure; (3) Cases with incomplete patient identifiers, such as no inpatient and outpatient numbers or errors.

### Data collection

**Fig. 1:**
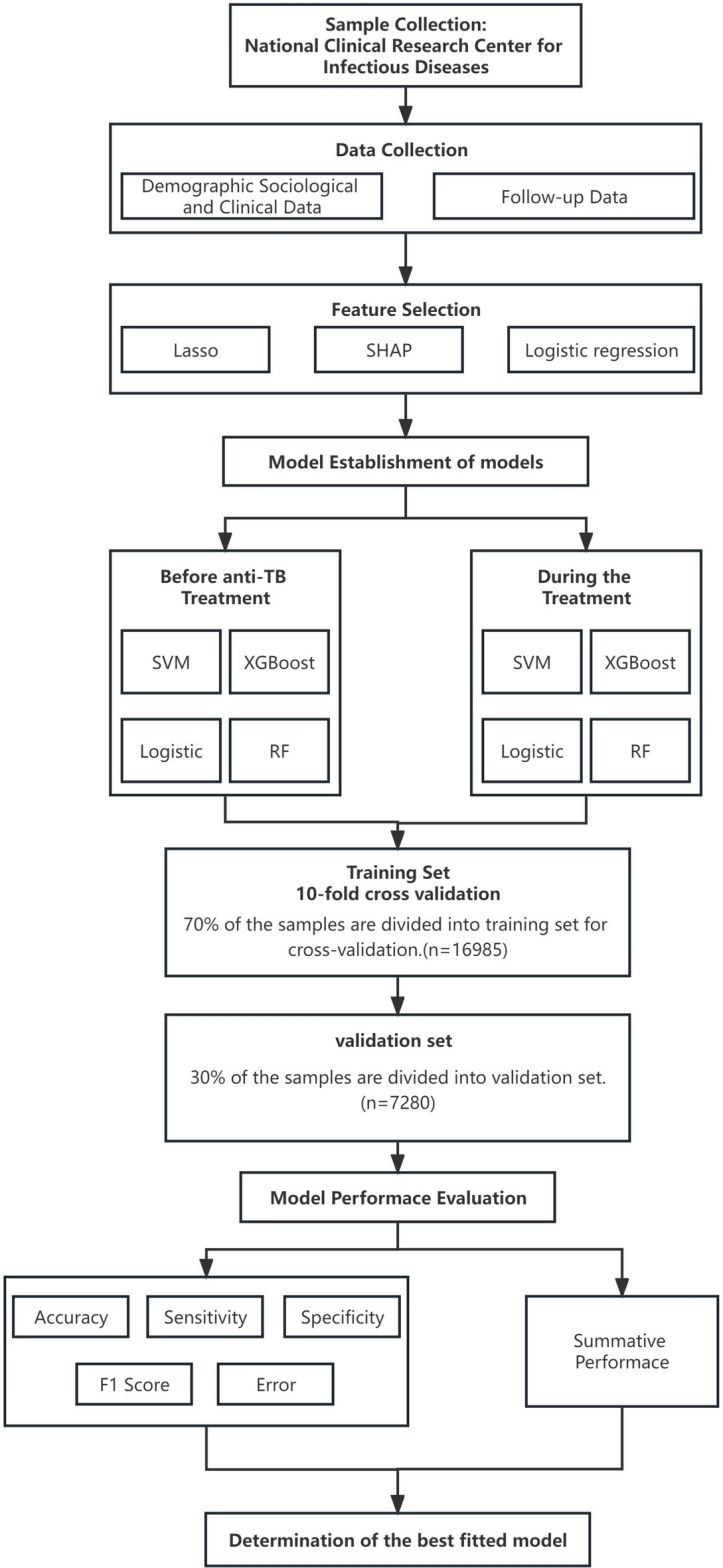
Study flowchart.

Clinical data were obtained from the Hospital Information System (HIS), Laboratory Information System (LIS), Picture Archiving and Communication System (PACS), and Shenzhen Chronic Disease Follow-up Management System (SCDFMS) of the Shenzhen Third People’s Hospital. The Hospital Information System and the Chronic Disease Follow-up Management System are two separate information systems that operate independently. Therefore, in order to integrate all the medical and follow-up management data of patients, this study used the hospitalization number and outpatient number for patient information matching, and the processing process used Python V3.9.7 to call packages such as pandas and numpy for the initial integration of patient data, and two-person review of the matched data and saving the data as files in .csv formats (Fig. 1).

### Feature selection variables

We drop the null value variables of the raw data to eliminate useless information, after which 35 variables were left three categories: (1) general patient information: name, age, gender, ethnicity, marital status, education, occupation, weight, BMI, household registration, and health insurance status, etc.; and (2) clinical medical care information: route of admission, mode of admission, year of diagnosis, clinical diagnosis, whether or not hospitalization was performed, bacteriological diagnosis, month of treatment, whether or not the patient had comorbid conditions such as hypertension, diabetes mellitus, chronic hepatitis or liver cirrhosis, or cancer, adverse reactions to anti-tuberculosis medication, history of smoking, history of drinking, psychological status, and self-care ability score; (3) Patient follow-up information: whether death occurred, time of death, patient-reporting hospital, whether treatment failed, time of treatment failure, and admission status.

### Statistical analysis

Mean values were used for normally distributed continuous variables, while categorical variables were described using values and proportions. Chi-square tests, t-tests, and logistic regression were used for between-group comparisons. We selected variables with non-zero coefficients in the least absolute shrinkage and selection operator (LASSO) analysis for further analysis. Variables with *P* < 0.05 in univariate logistic regression analyses were included in multivariate logistic regression analyses to identify independent risk factors. Variables with high significance were screened by SHapley Additive exPlanations (SHAP) values, and the results of the LASSO and regression analyses were combined to determine the final variables to be included in the model. Multivariate logistic algorithms such as RF, SVM, XGB and logistic regression were used to build the model. XGBoost is a gradient boosting algorithm that integrates multiple decision trees, improving on traditional gradient tree boosting techniques. Describe the results of tenfold cross-validation with a line graph. The area under the receiver operating characteristic curve (ROC) (AUC) is calculated, which is the most commonly used parameter to measure the summed performance of the model. Sensitivity, specificity and accuracy were also calculated to evaluate and compare the performance of all predictive models. All machine learning models were constructed and validated in R (version 4.2.1).

## Results

### Basic characteristics

This study analyzed a cohort of 24,265 tuberculosis patients, with 3036 (12.51%) experiencing LTFU. LTFU occurred in 2.79% (n = 678) of patients before treatment and 9.72% (n = 2358) during treatment. Among those LTFU before treatment, 66.5% were male, with an average age of 40.9 ± 17.0 years, and 60.2% had only primary education or less. During treatment, 64.4% of the 2358 LTFU patients were male, 68.2% were married, and 20.0% had primary education or less. Employment status showed slight variations, with 54.8% of those LTFU before treatment being employed compared to 52.5% during treatment. Smoking and drinking habits were more prevalent among those LTFU before treatment, with 15.5% and 28.0% respectively, compared to 11.2% and 17.5% during treatment. Additionally, health insurance coverage was slightly lower in the LTFU group both before (67.1%) and during treatment (66.5%) compared to those who did not experience LTFU (Table 1).

**Table 1:** Characters of TB patients with LTFU label before the treatment.

Variables	Category	Before treatment initiation		*P*-value	After treatment initiation		*P*-value
		Non-LTFU n = 23,587 (%)	LTFU n = 678 (%)		Non-LTFU n = 21,219 (%)	LTFU n = 2368 (%)	
Sex	Male	15,444 (65.5)	445 (66.5)	0.568	13,913(65.6)	1529(64.4)	0.234
	Female	8152(34.5)	224(33.5)		7305(34.4)	847(35.6)	
Ethnicity	Han	23,160(98.2)	604(90.3)	< 0.001	20,840(98.2)	2320(97.6)	0.052
	Others	436(1.8)	65(9.7)		380(1.8)	56(2.4)	
Education	Primary School or under	5028(21.3)	403(60.2)	< 0.001	4552(21.5)	476(20.0)	0.206
	Junior	5434(23.0)	57(8.5)		4896(23.1)	538(22.6)	
	Senior	7991(33.5)	160(23.9)		7072(33.3)	839(35.3)	
	Associate Degree	2838(12.0)	30(4.5)		2542(12.0)	296(12.5)	
	Bachelor’s Degree and above	2385(10.1)	19(2.9)		2158(10.2)	227(9.6)	
Marital status	Married	16,217 (68.7)	452 (67.6)	0.522	14,595 (68.8)	1622 (68.2)	0.609
	Single/Divorced /Widowed	7397(31.3)	217(32.4)		6625(31.2)	754(31.7)	
Occupation	Employee	10,079 (50.0)	319 (54.8)	0.022	9018 (49.7)	1061 (52.5)	0.018
	Unemployed	9943(42.1)	259(39.5)		9265(42.2)	937(40.4)	
	Students	1490(6.3)	39(5.9)		1384(6.3)	145(6.2)	
	Civil	1061(4.4)	24(3.6)		969(4.4)	116(5.0)	
	Others	836(3.5)	20(3.0)		769(3.5)	87(3.7)	
Smoking	Yes	2619(11.1)	104(15.5)	< 0.001	2354(11.1)	265(11.2)	0.930
	No	20,977(88.9)	565(84.5)		18,866(88.9)	2111(88.8)	
Drinking	Yes	3474(14.7)	187(28.0)	< 0.001	3058(14.4)	416(17.5)	< 0.001
	No	20,122(85.3)	482(72.0)		18,162(85.6)	1960(82.5)	
Health insurance	Yes	16,836(71.4)	449 (67.1)	0.017	15,257(71.9)	1579 (66.5)	< 0.001
	No	6760(28.6)	220(32.9)		5963(28.1)	797(33.5)	
Residential status	Permanent	14,416(61.1)	412 (61.6)	0.798	12,999(61.3)	1417 (59.6)	0.124
	Temporary	9180(38.9)	257(38.4)		8221(38.7)	959(40.4)	
Residence registration	Urban	17,592(97.3)	492(2.7)	0.553	15,827(74.6)	1765(74.3)	0.750
	Rural	6004(25.4)	177(26.5)		5393(25.4)	611(25.7)	
	Hypertension	921(3.9)	28(4.2)	0.710	846(4.0)	75(3.2)	
Extrapulmonary TB	Yes	3198 (13.6)	98 (14.6)	0.415	2856 (13.5)	342 (14.4)	0.207
Underlying disease	Diabetes	1731 (7.3)	61 (9.1)	0.082	1540 (7.3)	191 (8.0)	0.166
	Chronic Hepatitis/Cirrhosis	1350 (5.7)	45 (6.7)	0.271	1185 (5.6)	165 (6.9)	0.007
	Cancer	302(1.3)	6(0.9)	0.383	269(1.3)	33(1.4)	
	No	20,398(86.4)	571(85.4)		18,364(86.5)	2034(85.6)	
RR/DR-TB	Yes	709 (3.0)	20 (3.0)	0.982	646 (3.0)	63 (2.7)	0.288
	No	22,887(97.0)	649(97.0)		20,574(97.0)	2313(97.3)	
Negative results of AFB smear/GeneXpert/Culture	Yes	10,907(46.2)	428 (64.0)	< 0.001	9326 (43.9)	1581 (66.5)	< 0.001
	No	12,689(53.8)	241(36.0)		11,894(56.1)	795(33.5)	
Previous history of TB	Yes	1163 (4.9)	46 (6.9)	0.022	1042 (4.9)	121 (5.1)	0.697
	No	22,433(95.1)	623(93.1)		20,178(95.1)	2255(94.9)	
Had been hospitalized	Yes	15,284(64.8)	131 (13.1)	< 0.001	14,809(69.8)	475 (20.0)	< 0.001
	No	20,398(86.4)	571(85.4)		18,364(86.5)	2034(85.6)	
RR/DR-TB	Yes	709 (3.0)	20 (3.0)	0.982	646 (3.0)	63 (2.7)	0.288
	No	22,887(97.0)	649(97.0)		20,574(97.0)	2313(97.3)	
Negative results of AFB smear/GeneXpert/Culture	Yes	10,907(46.2)	428 (64.0)	< 0.001	9326 (43.9)	1581 (66.5)	< 0.001
	No	12,689(53.8)	241(36.0)		11,894(56.1)	795(33.5)	
Previous history of TB	Yes	1163 (4.9)	46 (6.9)	0.022	1042 (4.9)	121 (5.1)	0.697
	No	22,433(95.1)	623(93.1)		20,178(95.1)	2255(94.9)	
Had been hospitalized	Yes	15,284(64.8)	131 (13.1)	< 0.001	14,809(69.8)	475 (20.0)	< 0.001
	No	8312(35.2)	538(80.4)		6411(30.2)	1901(80.0)	
Provision of an alternative contact	Yes	19,525(82.7)	546 (81.6)	0.445	17,611(83.0)	1914(80.6)	0.003
	No	4071(17.3)	123(18.4)		3609(17.0)	462(19.4)	
Admission method	Emergency	8687 (36.8)	511 (76.4)	< 0.001	6936 (32.7)	1751(73.7)	< 0.001
	Outpatient	14,909(63.2)	158(23.6)		14,284(67.3)	625(26.3)	
Occurrence of Adverse drug reaction	Yes	–	–		1222 (5.8)	183 (7.7)	< 0.001
	No	–	–		19,998(94.2)	2193(93.2)	
Age (Median [IQR])	–	41.3 ± 17.0	40.9 ± 17.0	0.502	41.3 ± 17.0	40.1 ± 16.9	0.248
Weight, kg	–	56.1 ± 7.2	56.8 ± 5.4	0.006	56.04 ± 7.2	56.16 ± 7.6	0.454
BMI, kg/m^2^	–	20.5 ± 2.2	20.5 ± 1.6	0.973	20.5 ± 2.2	20.5 ± 2.3	0.500
ADL score	–	90.0 ± 13.5	89.8 ± 10.7	0.620	90.0 ± 13.5	90.2 ± 13.4	0.647

A preliminary univariate analysis, detailed in Table 1, was conducted to select relevant risk factors. This analysis revealed associations between LTFU before treatment and factors including education, ethnicity, smoking, drinking, occupation, health insurance, weight, history of hospitalization, and admission method. Comparing the non-LTFU and LTFU groups during treatment, notable intergroup disparities surfaced in ethnicity, drinking habits, occupation, health insurance status, underlying medical conditions, negative outcomes in smear/GeneXpert/culture tests, history of hospitalization, provision of alternative contacts, admission methods, and occurrences of adverse drug reactions (Table 1).

### Predictor variable selection of LTFU

**Fig. 2:**
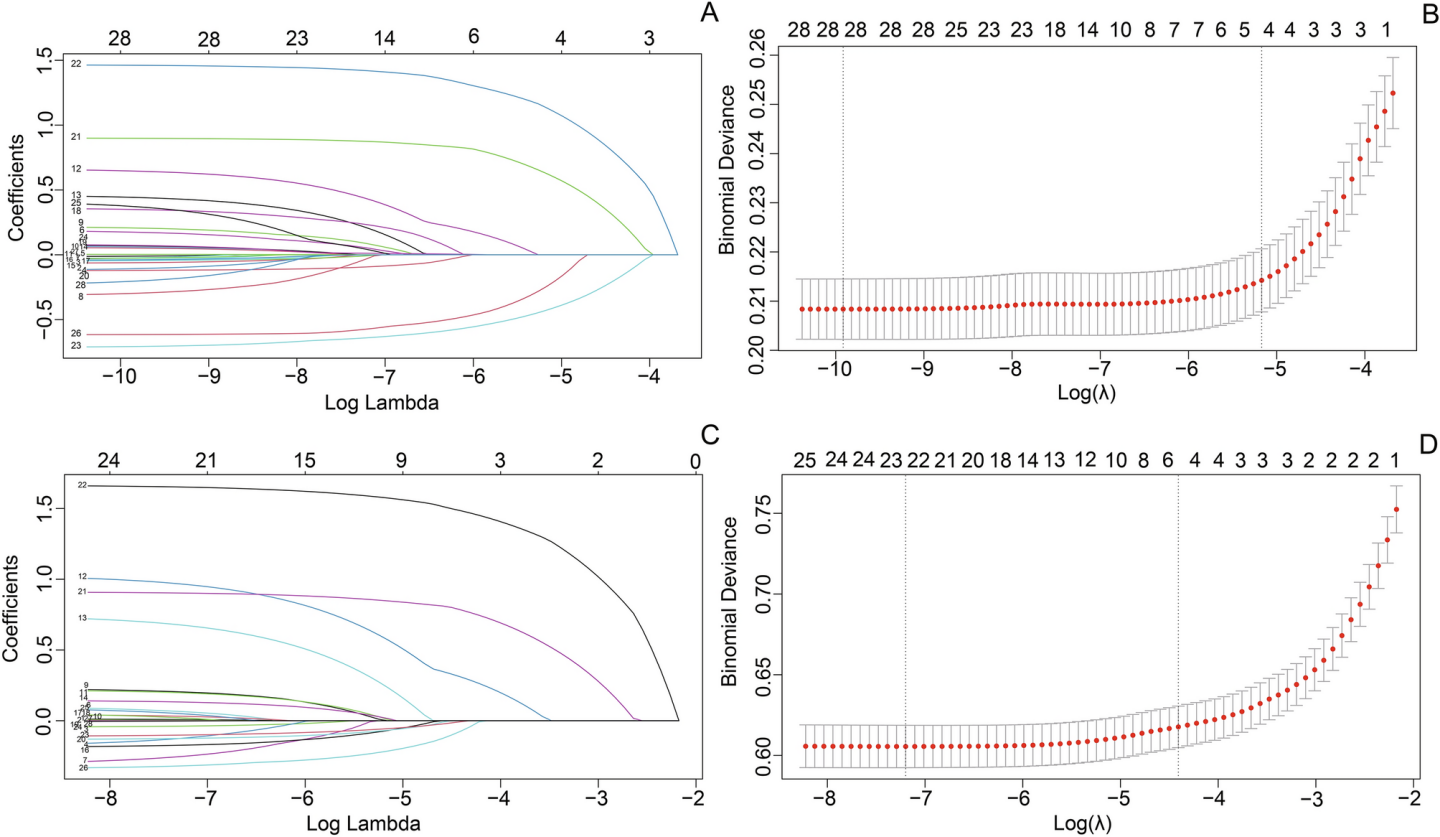
Variable selection using LASSO method. (**A** and **C**) A coefficient profile plot. evertical axis represents the coefficients, and the horizontal axis represents log (lambda). (**B** and **D**) A binomial deviance curve.

Univariate analyses identified predictive variables associated with LTFU before and during treatment. For the period preceding treatment initiation, protective factors included various educational levels, including junior high (aOR 0.12, 95% CI 0.09–0.17), senior high school (aOR 0.27, 95% CI 0.22–0.34), associate degree (aOR 0.15, 95% CI 0.10–0.22), and undergraduate (aOR 0.10, 95% CI 0.06–0.17), alongside a history of hospitalization (aOR 0.22, 95% CI 0.17–0.28). Conversely, being employed (aOR 1.21, 95% CI 1.02–1.43), engaging in alcohol consumption (aOR 2.18, 95% CI 1.79–2.65), outpatient admission (aOR 2.18, 95% CI 1.79–2.65), and having a prior history of tuberculosis (aOR 1.64, 95% CI 1.18–2.29) emerged as independent risk factors for LTFU before treatment. During the treatment phase, influential factors encompassed employment status (aOR 1.15, 95% CI 1.04–1.27), history of hospitalization (aOR 0.19, 95% CI 0.17–0.22), outpatient admission (aOR 2.53, 95% CI 2.25–2.85), presence of chronic hepatitis/cirrhosis (aOR 1.26, 95% CI 1.04–1.53), experiencing drug adverse reactions (aOR 1.24, 95% CI 1.02–1.50), availability of an alternative contact (aOR 0.82, 95% CI 0.72–0.94), and health insurance coverage (aOR 0.76, 95% CI 0.69–0.85). The Lasso regression and SHAP algorithm identified that BMI and smoking served as predictive factors for LTFU prior to treatment, while drinking proved significant in constructing the risk model for LTFU during treatment (Fig. 2 and Supplementary Fig. S1 and Table S1).

### Prediction by SVM, Logistic, XGBoost and RF

**Fig. 3:**
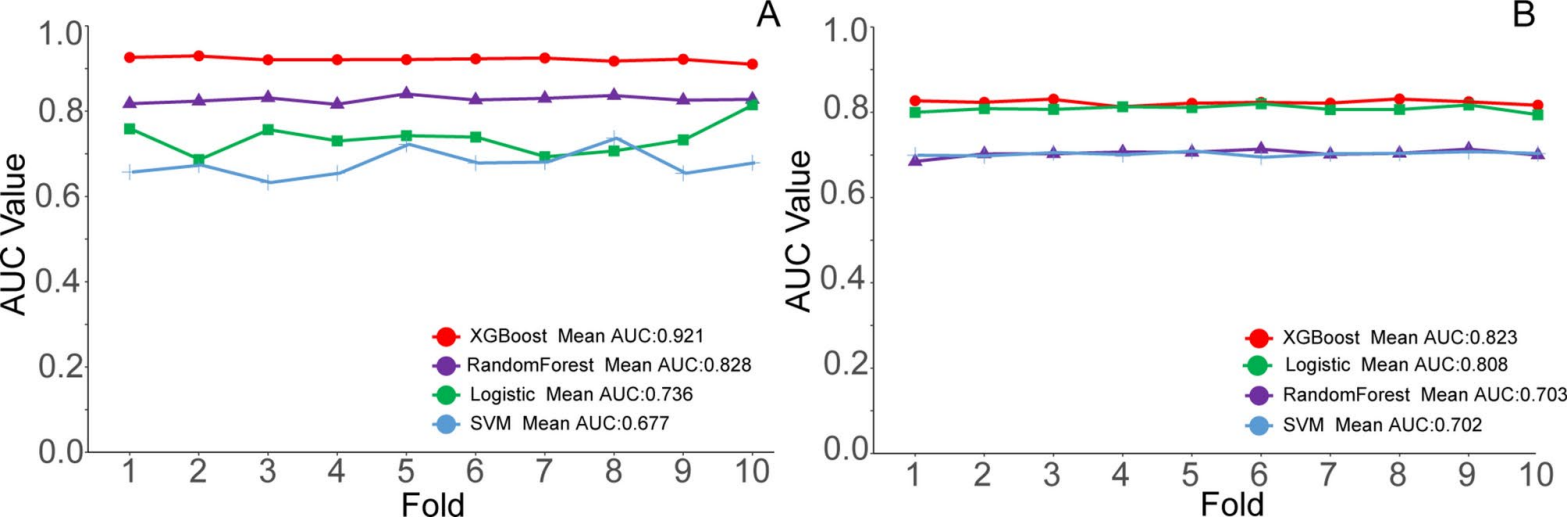
Ten-foldcross-validation of 4 ML algorithms for predicting LTFU before and after the initiation.

For the prediction models assessing the risk of LTFU before the treatment initiation, the outcomes of tenfold cross-validation demonstrated consistent superiority of XGBoost over the other three models. XGBoost achieved the highest average AUC (0.921), outperforming Random Forest (0.828), Logistic Regression (0.736), and SVM (0.677), demonstrating its superior predictive capability for LTFU before treatment (Fig. 3).

In the validation set, XGBoost showed the highest sensitivity (0.81), F1 score (0.85), and AUC (0.921). The descending sequence of performance for the other models was Logistic (0.811), RF (0.755), and SVM (0.712). Remarkably, both XGBoost and RF models yielded the same F1 score of 0.75 among the models addressing LTFU during the treatment. Notably, XGBoost demonstrated the highest AUC value (0.825), surpassing Logistic (0.818), RF (0.692), and SVM (0.688) (Table 2).

**Table 2:** Validation outcomes of the models.

	AUC	Accuracy	Sensitivity	Specificity	F1 score
Logistic(A)	0.811	0.79	0.64	0.88	0.77
RF(A)	0.755	0.85	0.76	0.90	0.83
XGBoost(A)	0.921	0.87	0.81	0.91	0.85
SVM(A)	0.712	0.79	0.72	0.82	0.73
Logistic(B)	0.818	0.73	0.59	0.82	0.69
RF(B)	0.692	0.76	0.55	0.88	0.75
XGBoost(B)	0.825	0.76	0.53	0.89	0.75
SVM(B)	0.688	0.76	0.74	0.77	0.62

*(A) The model for LTFU before treatment in tuberculosis patients, (B) The model for LTFU during the treatment period.

## Discussion

Tuberculosis (TB) remains a global health challenge, with fatalities rising from 1.4 million in 2019 to 1.6 million in 2021^1^. A significant contributor to TB mortality is the loss of follow-up (LTFU), highlighting the need for effective disease management^2^. However, there has been limited exploration of this issue among Chinese patients Thus, This study aims to address this gap by using retrospective data to develop a risk prediction model. Our goal is to identify key determinants and provide insights to improve TB management, enhance treatment adherence, and elevate patient outcomes in China.

This study uses advanced machine learning, particularly XGBoost, to predict treatment interruption in TB patients. This approach is essential in the big data era, where traditional methods may fall short. Compared to previous studies, our XGBoost model demonstrated significantly higher predictive accuracy. Rodrigo et al. used logistic regression for predicting lost visits in TB patients, achieving an AUC of 0.67^14^. whereas our model achieved an AUC of 0.921 for pre-treatment LTFU and 0.825 for in-treatment LTFU. Similarly, Hokino Yamaguti et al. developed a classification tree model with an accuracy of 0.76 and an F1 score of 0.77^15^, both of which were outperformed by our XGBoost model. These comparisons underscore the relevance of our study, as it demonstrates the superiority of machine learning techniques like XGBoost in handling complex variable relationships and improving prediction accuracy in the context of TB treatment interruption. XGBoost’s robustness comes from its ability to handle complex nonlinear relationships, fast training, and efficient memory usage, making it ideal for large healthcare datasets. Additionally, its gradient boosting framework ensures high accuracy even with small sample sizes, a crucial advantage in healthcare^16^.

Machine learning, particularly XGBoost, has proven highly effective in capturing complex data patterns, as demonstrated by our study. This approach significantly enhances prediction accuracy in TB treatment outcomes, offering a robust tool for addressing the challenges of treatment interruption. The superior performance of the XGBoost model in predicting treatment interruption risk for TB patients aligns with previous research showcasing its remarkable capabilities in various prediction tasks^17^. Our study further validates the superiority of the XGBoost model over SVM, RF, and logistic regression models in predicting treatment interruption risk, highlighting its prowess in handling complex variable relationships^18^. Our study contributes to the growing evidence supporting XGBoost as a potent tool in healthcare prediction tasks. Its versatility in medical scenarios makes it an invaluable asset for healthcare professionals seeking accurate risk assessments. Future research should assess the generalizability of our findings across diverse populations, enhancing the applicability of XGBoost. High-performance models like XGBoost offer a proactive strategy to reduce treatment interruption and improve TB treatment success rates. XGBoost accurately identifies high-risk patients, enabling efficient resource allocation, reducing workload, and improving patient care. This personalized approach significantly lowers treatment interruption rates, optimizing TB management. As a result, our study offers insightful strategies for data-driven approaches in TB treatment and underscores the potential of XGBoost as a valuable tool in predictive healthcare analytics.

We identified key risk factors for TB patient dropout before treatment, with outpatient clinic patients showing higher LTFU rates. Consequently, tailoring educational efforts towards patients who have experienced relapses and are attending outpatient clinics assumes paramount importance in augmenting treatment adherence. A notable revelation from our study was the independent association of alcohol consumption with TB patient dropout the trend that aligns with findings from a prospective study in Ethiopia^19^, this underscores the urgency of providing meticulous guidance to patients who consume alcohol, encouraging strict adherence to medication regimens throughout the TB treatment process. Our study’s insights underscore the significance of addressing specific risk factors like a history of TB and alcohol consumption, providing avenues for curbing treatment dropout in TB patients. Targeted interventions and enhanced patient education could improve treatment adherence, leading to better outcomes and reduced disease burden.

The inclusion of hospitalized TB patients in our study demonstrated a noteworthy decrease in the risk of LTFU. Hospitalization of TB patients exhibited a notable reduction in the risk of treatment interruption. Hospitalization provided advantageous conditions for treatment adherence by facilitating interaction with healthcare professionals, delivering health education, and ensuring continuous medical care until discharge criteria were met. This vigilant supervision and support during hospitalization contributed to enhanced adherence rates^20^, while the gradual recovery process during hospital stay instilled patient confidence in maintaining their treatment regimen^21^. These findings underscore the pivotal role of hospitalization, particularly in urgent cases, in fortifying treatment adherence for TB patients. Moreover, educational attainment emerged as a significant factor in reducing the likelihood of treatment interruption among TB patients. Patients with higher educational backgrounds often possess a more profound understanding of health concepts, heightened health awareness, and comprehensive knowledge of their health status and treatment options^22^. This heightened awareness drives them to prioritize TB management and treatment, recognizing the value of prompt medical attention and strict adherence to prescribed treatment protocols. Thus, targeted educational initiatives tailored to patients with lower educational levels can effectively enhance TB awareness, underscoring the significance of both treatment adherence and timely medical intervention.

Interestingly, chronic hepatitis or cirrhosis emerged as a unique risk factor for treatment interruption during antituberculosis treatment, yet intriguingly, it did not exert the same influence on treatment interruption before treatment initiation. This divergence can be attributed to the heightened vulnerability of patients with chronic liver disease to hepatotoxicity during antituberculosis treatment, prompting preemptive treatment discontinuation to prevent further liver damage^23^. Additionally, our study highlights that patients who experience adverse effects are more susceptible to treatment interruption after initiating therapy. Severe drug side effects compel patients to discontinue treatment, and frequent changes in the treatment regimen due to adverse reactions can test patients’ resilience, ultimately leading to treatment discontinuation and interruptions in follow-up^24^. Hence, enhancing the training of medical personnel in effectively monitoring and managing adverse drug reactions becomes crucial. Establishing a comprehensive framework for reporting and managing adverse drug reactions can assist patients in overcoming treatment challenges and reinforcing treatment adherence. Moreover, independent employment status emerged as a risk factor for treatment interruption, a finding contrasting with prior research associating unemployment with such interruptions^25^. Our hypothesis suggests that a substantial proportion of employed or self-employed individuals might experience social and occupational pressures that contribute to a negative impact and elevate the risk of LTFU.

The registration of backup contact information plays a crucial role in mitigating the risk of treatment interruption during antituberculosis therapy. Typically, these backup contacts, often family members, provide essential support to healthcare providers by facilitating communication, scheduling follow-up appointments, and ensuring adherence to treatment plans. Furthermore, meticulous documentation of patient information throughout treatment holds significant value, aiding in patient management, treatment decision-making, and providing invaluable data to support local TB elimination strategies^26^. Additionally, the presence of health insurance emerges as a protective factor against treatment interruption. Our study underscores previous research by providing compelling evidence that patients lacking health insurance face a higher risk of poor treatment outcomes^27^. The absence of health insurance leads to increased medical expenses. In alignment with Article 21 of the Measures of the People’s Republic of China on the Management of Tuberculosis, TB patients are entitled to health insurance coverage for examinations and medication during treatment, with both national and local governments offering subsidies and exemptions for medical expenses related to tuberculosis treatment^28^. Considering the association between TB and poverty, effective management interventions should prioritize alleviating the economic burden faced by TB patients.

While this study offers valuable insights, some limitations should be noted. We analyzed LTFU separately before and during treatment, which provides stage-specific insights but may not capture the overall impact of LTFU. Additionally, we did not compare binary outcomes of LTFU versus non-LTFU across the entire cohort, nor did we conduct a ternary analysis of non-LTFU, LTFU before treatment, and LTFU during treatment, which could highlight different epidemiological factors. The duration and reasons for hospitalization were also not explored in depth, which may influence treatment outcomes. Despite these limitations, our findings contribute significantly to understanding the factors influencing LTFU in TB patients and provide a solid foundation for future research to build upon.

## Conclusion

In conclusion, our study highlights the superior predictive power of the XGBoost model in forecasting LTFU among TB patients, outperforming traditional machine learning methods. The identification of key risk factors, such as a history of previous tuberculosis, alcohol consumption, and lower educational attainment, offers valuable guidance for designing targeted interventions to enhance treatment adherence. Additionally, hospitalization was identified as a crucial factor in reducing the risk of LTFU, further emphasizing the importance of continuous care. By employing advanced machine learning techniques like XGBoost, we can more effectively address LTFU, thereby improving TB treatment success rates and patient outcomes. Our research advances the development of data-driven strategies in TB management and supports the implementation of personalized interventions to mitigate the impact of LTFU and optimize patient care.

## Supplementary Information

Supplementary Information.

## Data Availability

The datasets used and/or analyzed during the current study are not publicly available due to privacy concerns and institutional regulations regarding the handling of patient data. However, the datasets are available from the corresponding author on reasonable request. Requests for access to the data should include a clear justification, and access will be granted in accordance with relevant ethical guidelines and institutional policies.
