# Supplementary material for: Predictive machine learning models for anticipating loss to follow-up in tuberculosis patients throughout anti-TB treatment journey
Source: Sci Rep. 2024 Oct 21;14:24685. doi: 10.1038/s41598-024-74942-z (PMC11494039; doi:10.1038/s41598-024-74942-z)
Supplement: Supplementary file 1 — Supplementary Information. [file 41598_2024_74942_MOESM1_ESM.docx]

**TbaleS1. Multivariate logistic regression for patients with LTFU label before the treatment or during the treatment.**

| Variables | Category | LTFU before the initiation | | LTFU after the initiation | |
| --- | --- | --- | --- | --- | --- |
|  |  | Adjusted odds  ratio (95% CI) | P-value | Adjusted odds  ratio (95% CI) | P-value |
| Education | Junior（*vs* Primary School or under） | 0.12(0.09–0.17) | <0.001 |  |  |
|  | Senior（*vs* Primary School or under） | 0.27(0.22-0.34) | <0.001 |  |  |
|  | Associate Degree（*vs* Primary School or under） | 0.15(0.10-0.22) | <0.001 |  |  |
|  | Bachelor's degree and above（*vs* Primary School or under） | 0.10(0.06-0.17) | <0.001 |  |  |
| occupation | Employee （*vs* Unemployed） | 1.21(1.02–1.43) | 0.029 | 1.15(1.04–1.27) | 0.005 |
| Drinking | Yes（*vs* No） | 2.18(1.79-2.65) | <0.001 |  |  |
| Has been hospitalized | Yes（*vs* No） | 0.22(0.17–0.28) | <0.001 | 0.19(0.17–0.22) | <0.001 |
| Admission method | Outpatient（*vs* Emergency) | 2.61(2.10–3.25) | <0.001 | 2.53(2.25-2.85) | <0.001 |
| Previous history  of TB | Yes（*vs* No） | 1.64(1.18–2.29) | 0.004 |  |  |

**
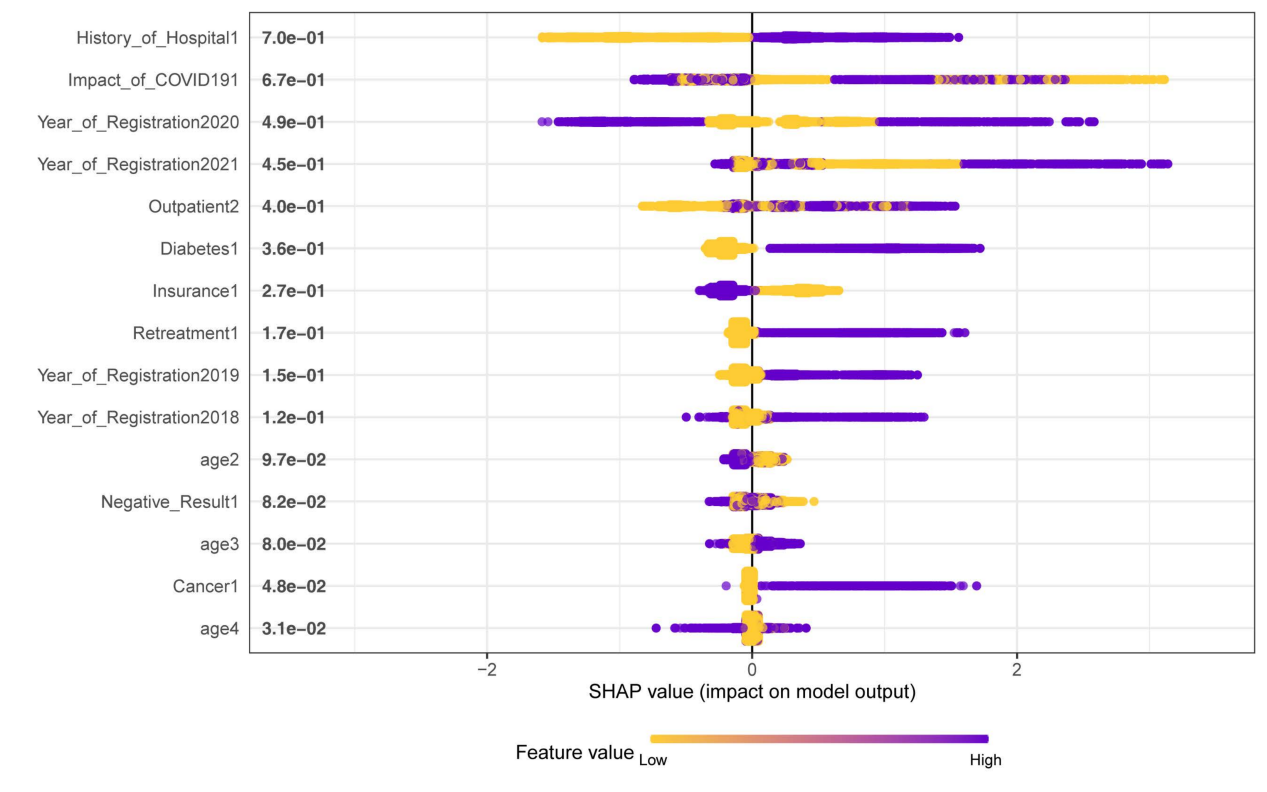
**

**Supplementary figure S1.1. SHAP value of LTFU before the treatment.**

**
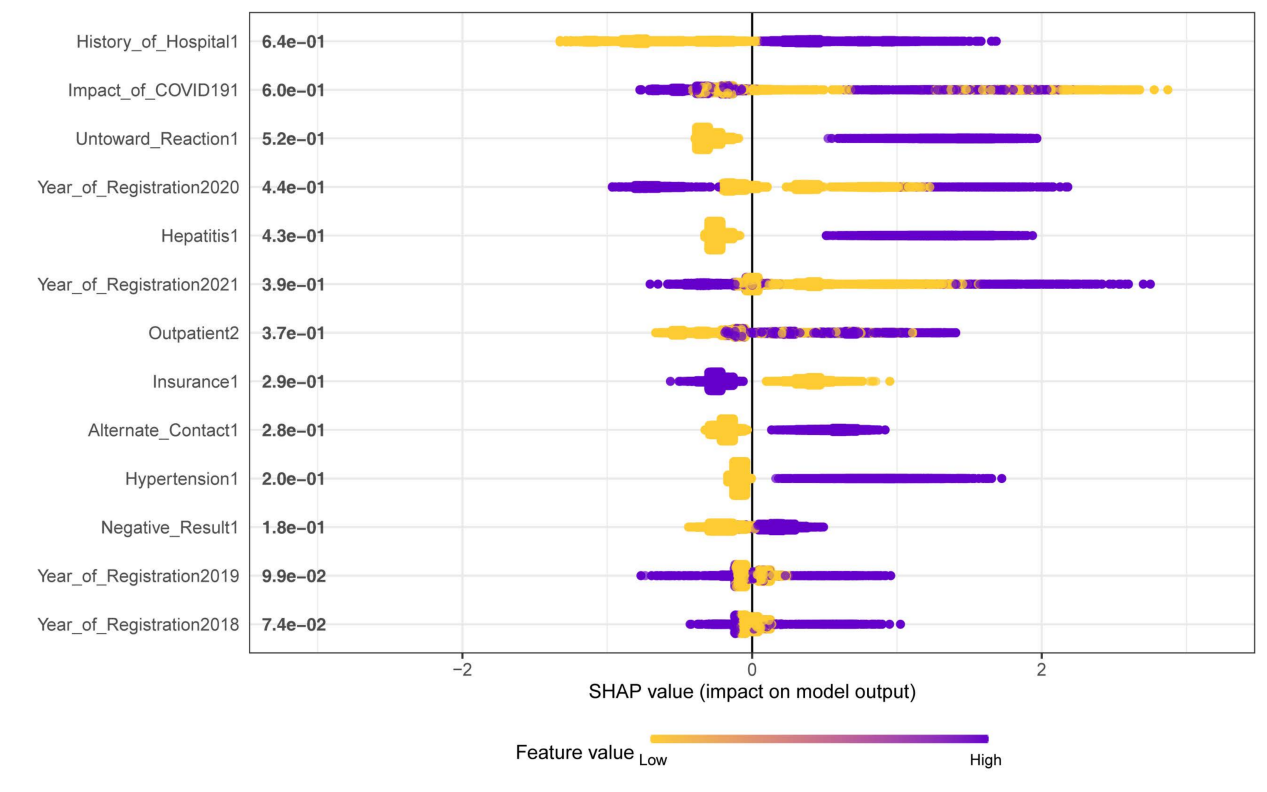
**

**Supplementary figure S1.2. SHAP value of LTFU during the treatment.**
